# Common drivers of seasonal movements on the migration – residency behavior continuum in a large herbivore

**DOI:** 10.1038/s41598-018-25777-y

**Published:** 2018-05-16

**Authors:** Jodie Martin, Vincent Tolon, Nicolas Morellet, Hugues Santin-Janin, Alain Licoppe, Claude Fischer, Jérôme Bombois, Patrick Patthey, Elias Pesenti, Delphine Chenesseau, Sonia Saïd

**Affiliations:** 1 Office national de la chasse et de la faune sauvage, Unité ongulés sauvages, 01330 Birieux, France; 2 Centre for African Ecology, School of Animal, Plant and Environmental Sciences, University of the Witwatersrand, Johannesburg, South Africa; 3 ISARA-Lyon 23 rue Jean Baldassini, Lyon, 69007 France; 4 CEFS, Université de Toulouse, INRA, Castanet-Tolosan, France; 5 Office National de la Chasse et de la Faune Sauvage, 39 Bd Albert Einstein, 44323 Nantes, Cedex 3 France; 6 Venn Life Sciences, 63 bd Haussman, 75008 Paris, France; 7 Département de l’Etude du Milieu naturel et agricole, Service public de Wallonie, Gembloux, Belgium; 8 University of Applied Sciences of Western Switzerland, 1274 Jussy, Switzerland; 9 Fédération départementale des chasseurs du Jura, Rue de la fontaine salée, 39140 Arlay, France; 10 Direction générale de l’environnement, Ch. Du Marquisat 1, CH – 1025 St Sulpice, Switzerland; 11 Service des forêts et de la faune, Route du Mont Carmel 1, Case postale 155, 1762 Givisiez, Switzerland; 12 Office national de la chasse et de la faune sauvage, Délégation interrégionale Bourgogne Franche-Comté, 57 rue de Mulhouse, 21000 Dijon, France

## Abstract

This study aimed to (1) identify the scale of environmental drivers of seasonal movements on the migration – residency behavior continuum in a large herbivore species and to (2) test the hypothesis that the same environmental drivers and spatio-temporal scaling should influence spatial processes in both migrants (long distance migration) and residents (short distance range shifts). We performed a comparative analysis of the influence of plant phenology and snow cover duration on seasonal movements of five partially migrating red deer populations with contrasting environmental conditions, at the seasonal range scale and at the study area scale. The five populations presented varying proportions of migrants, large gradients of migration distances and seasonal range shifts. The probability for a red deer to migrate was strongly influenced by large-scale environmental conditions, consistent with the resource heterogeneity hypothesis (high spatio-temporal scaling favors migration). Distances moved by both migrants and residents were strongly related to large-scale environmental conditions as well. We showed that similar proximal causes influenced these seasonal movements, reinforcing the idea of a continuum from migration to residency in response to seasonal environmental changes. Together, our findings suggest that global warming, by homogenizing large-scale environmental conditions, may thus decrease migratory tactics.

## Introduction

In recent years, a decline of migratory animals has been observed, for different taxa in different biomes^1,2^. Global change is one of the non-exclusive causes identified as responsible for this threat^2^. In particular, the global warming has been shown to negatively affect migratory populations as shifting climatic conditions may cause a mismatch between the cues that trigger migration and actual environmental conditions at the arrival range^3,4^. To predict the sensitivity of migratory species to environmental change, we need to identify the causative relationship between environmental variability and population movements. Identifying first the proximal causes of migration movements is thus a necessary step to better understand animal response to climate change and implement better conservation strategies.

Partial migration, where both migrant and resident tactics co-occur within a population, has been described as a behavioral continuum between one-trip migration and residency and is common in birds^5,6^, fish^7,8^ and mammals^9,10^. Migrants are usually defined as individuals with non-overlapping seasonal home-ranges^9,11–14^. The partitioning of individuals into migrant and resident tactics is always the starting point to further investigate variation in migrant and migration characteristics, considering that residents are strictly sedentary (overlapping seasonal home-ranges) and excluding them from further investigation. However, residents may express range contraction/expansion or shifts between seasons^15,16^ and few study have simultaneously investigated factors influencing seasonal movements of both migrants and residents in a common framework. While much behavioral research has focused on understanding drivers and triggers of migration, our understanding of spatial dynamic of partially migrating populations thus remains incomplete.

In highly seasonal and heterogeneous environments, partial migration is commonly observed in large herbivores (e.g. roe deer (*Capreolus capreolus*)^9,17^; red deer (*Cervus elaphus*)^18,19^; moose (*Alces alces*)^20^) in order to exploit spatio-temporal changes in resource quality and quantity^11,21^. Migration movements has thus recently received a lot of attention in terms of both conceptual and methodological aspects^9,19,21–24^. Among the non-exclusive factors driving migration, environmental conditions are the main factors^18,25^. In northern latitudes, plant phenology and snow in particular have been shown to trigger migratory movements^19^. The forage maturation hypothesis (FMH), which states that large herbivores should track high forage quality and should profitably exploit the geographic gradients in resources, has received support in explaining migratory characteristics of large herbivores^18,21^. Migration thus should benefit populations living in areas with large-scale spatio-temporal heterogeneity in resources, as contrasting plant phenology or snow cover duration/depth between remote areas should promote long-distance home-range shifts. Individuals should thus remain resident in static environments or when heterogeneity in resources occurs at fine spatial scale, while they should migrate in spatio-temporally dynamic environments when the variability is temporally predictable, i.e. in environment with large-scale spatial heterogeneity and predictability of resource productivity^26^.

In this study, we consider both long-distance migration by migrant individuals and short-distance home-range shifts by resident individuals as a continuum in behavioral responses to seasonal change, hereafter called ‘seasonal movements’. In particular, our objective was to provide a better understanding of the environmental factors responsible for both long and short-distance seasonal movements, and to test the hypothesis that the same environmental factors should drive all seasonal movements. We thus determined (1) the environmental factors leading to higher proportion of migrant individuals in partially migratory populations and (2) how they affect both seasonal movement types (long and short movements). In addition to explaining the propensity to migrate, large-scale spatio-temporal heterogeneity of resources productivity has been shown to influence the distances moved by migrants^23,24,26,27^. While long distances are correlated to large-scale heterogeneity of resource productivity, fine-scale spatial heterogeneity induces short distance movements or residency^24^.

We thus explored the influence of plant phenology and snow cover duration (hereafter called ‘environmental conditions’) on seasonal movements at two different spatial scales: the seasonal range scale (hereafter referred to as ‘local-scale’) and the study area scale (hereafter referred to as ‘large-scale’). We performed a comparative analysis of factors influencing both migration and seasonal range shifts of resident individuals in five populations of red deer (*Cervus elaphus*) located along a latitudinal and altitudinal gradient and with varying environmental conditions. More specifically, we tested the influence of spatial scaling (large- *versus* local-scale) of snow duration and plant phenology (greenup dates for spring and senescence dates for autumn), at the population scale, on (1) the probability to migrate, (2) the distances moved for migrants and (3) the seasonal range shifts for residents. We analyzed autumn and spring movements separately to investigate specific drivers influencing seasonal movements from winter to summer ranges (in spring) and summer to winter ranges (in autumn) independently.

First, following the ‘resource heterogeneity hypothesis’, we hypothesized that (H1) migration should be more predominant in landscapes with large-scale heterogeneity of resource productivity. We predicted that large-scale heterogeneity in snow cover duration and plant phenology should thus promote migration (higher probability to migrate) more than local-scale conditions. In addition, we predicted that in spring, pronounced large-scale heterogeneity in greenup dates should favor migration (H1a). Indeed, following the FMH, large herbivores, and especially red deer, should track early plant phenology, where possible^28^. If there is no heterogeneity in greenup dates, the cost-benefit balance of migrating should be in disfavor of this behavior. In autumn, it has been shown that large herbivore migration is strongly influenced by snow conditions^29^. Large-scale heterogeneity in snow cover duration should thus favor migration more than plant phenology in autumn (H1b). We expected that red deer should seek for areas where snow cover duration is shorter. Under weak or no large-scale spatio-temporal differences in snow cover duration or under fine-scale variation in snow cover duration, red deer populations should favor residency as no benefit of true migration is expected. As a consequence of these processes, we should find more contrasted greenup/senescence dates and snow cover duration in winter and summer ranges of populations with a higher proportion of migrating individuals.

Second, under the assumption that long distance migration and residency are the endpoints of a behavioral continuum, we expected that the same environmental drivers and spatio-temporal scaling should influence spatial processes in both migrants and residents. We thus hypothesized that large-scale heterogeneity of resource productivity should affect distances moved by migrants and the seasonal home-range range shifts by residents more than local-scale resource productivity (H2). More specifically, an increase in large-scale spatial heterogeneity of resource productivity should increase the distances moved by individuals^23,24,27^. Particularly, we predicted that in spring, in areas with large amplitude and predictability of greenup dates the migration distances should increase as red deer should tend to exploit the earliest flush of new green vegetation (H2a) and seasonal range shifts in residents should increase as they should seek for food resources (H2b). In autumn, areas with a large amplitude and predictability of snow cover duration should favor long distance migration as red deer should seek for areas with shorter duration of snow cover (H2c). Similarly, low plant productivity associated with vegetation depletion and early snow fall should promote higher seasonal range shifts (H2d).

## Results

In the following, ‘migration distances’ refers to seasonal movements by migrants and ‘seasonal range shifts’ refers to seasonal movements by residents. We used the generic term ‘seasonal movements’ to refer to any movements (migration distances or seasonal range shifts). All the environmental variables used in the models are detailed in the Material and Methods section and summarized in Supplementary Table S1.

Following visual inspection of the net squared displacement (NSD) of each individual, among the 50 resident individual-years (seasonal range overlap > 0%) identified in spring, five were misclassified due to short excursions (see Supplementary Fig. S1) from one seasonal range to the next seasonal range. These individuals were actually migrants but with overlapping seasonal ranges slightly > 0%. Similarly, in autumn, among the 66 individual-years classified as residents, five were actually migrants (see Supplementary Fig. S1). Regarding migrant individuals monitored for more than two seasons (22/34 individuals), they all went back to their initial range (overlap between seasonal range i and seasonal range i + 2 ranging from 23% to 81%) except for two individuals that remained resident between seasonal range i + 1 and seasonal range i + 2.

**Figure 1:**
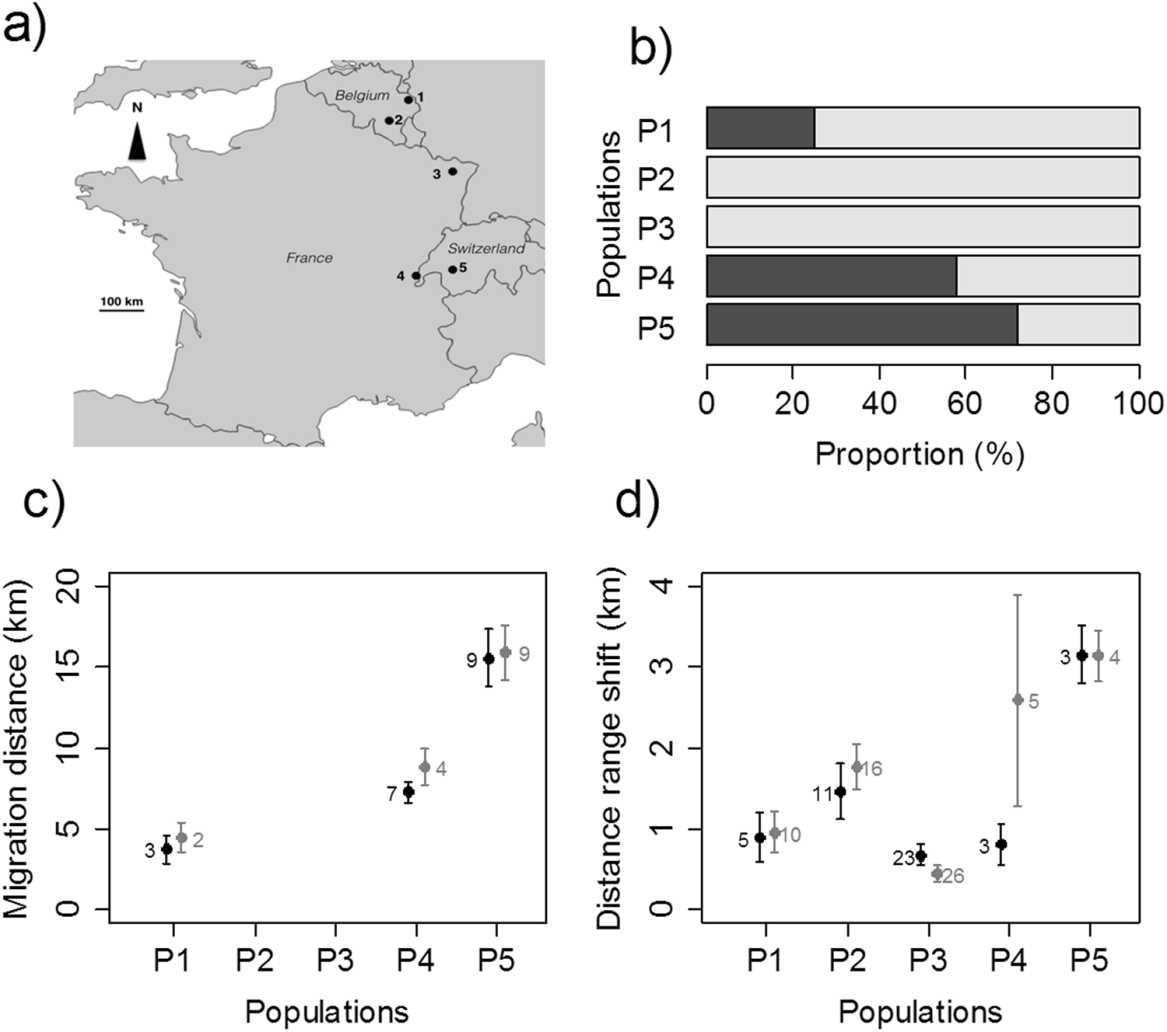
Location and large-scale movement patterns of five red deer populations. (**a**) Geographic locations of the five study areas, (**b**) proportion of migrants (dark gray) and residents (light gray) in each population, (**c**) mean and standard error of migration distances and (**d**) mean and standard deviation of seasonal range shifts for spring (black) and autumn (gray) migrations. Numbers nearby dots correspond to the number of individual-years for each migration season. The software QGIS 2.14 (https://www.qgis.org/fr/site/) and a shapefile of Europe freely available from http://www.arcgis.com/home/item.html?id=6d611f8d87d54227b494d4c3becef6a0 were used to create the figure in panel (a).

The five populations of red deer (see Table 1 for populations’ characteristics and names) presented varying proportions of migrants and residents and large gradients of migration distances and seasonal range shifts (Fig. 1). The proportion of migrating red deer ranged from 0% in P2 to 76% in P5. The migration distances varied from 4.08 ± 0.89 km (mean ± se) in P1 to 15.74 ± 1.73 km in P5. Seasonal range shifts for residents varied from 0.93 ± 0.28 km in P1 to 3.15 ± 0.34 km in P5. Seasonal home-range sizes were larger for migrant individuals (429.75 ± 42.37 ha; mean ± se) than for resident individuals (284.06 ± 15.14 ha; F = 17.00, p-value < 0.001), and larger in winter (409.14 ± 26.55 ha) than in summer (243.91 ± 15.22 ha; F = 34.98, p-value < 0.001). We could not conclude on differences in seasonal range sizes among populations (F = 2.53, p-value = 0.05). The ratio between the distances moved between seasons and the seasonal range sizes was higher for migrant individuals (F = 449, p-value < 0.001) and increased even more with the proportion of migrants in the population (F = 15.27, p-value < 0.001; see Table S2 in Supplementary information).

**Table 1:** Names and characteristics of the five study areas of red deer populations.

Populations	Lat/Lon	Elevation range (m)	Landscape characteristics	n	Study period
P1: Hertogenwald (Belgium)	50.5948/6.0221	250–450	Coniferous and mixed forest surrounded by crops and pastures.	7	2009–2013
P2: Saint Hubert (Belgium)	50.1027/5.3681	450–570	Deciduous and mixed forest surrounded by pastures.	10	2009–2013
P3: La Petite Pierre (France)	48.8321/7.3514	250–370	Mixed forest in nature reserve surrounded by crops and pastures.	35	2004–2013
P4: Jura Mountain (France – Switzerland)	46.4039/6.0304	400–1400	Coniferous and mixed forest surrounded by crops, pastures and alpine grassland at high elevation.	13	2012–2013
P5: Alpes (Switzerland)	46.5088/7.1530	800–1800	Coniferous and mixed forest patches within an alpine grassland and pasture matrix.	7	2009–2012

Lat/Lon = Latitude/Longitude in decimal degrees; n = number of individuals monitored in each study area.

*H1: Environmental factors affecting the probability to migrate*

Large-scale heterogeneity of environmental conditions, especially snow cover duration, influenced the probability to migrate more than local-scale environmental conditions, both for spring and autumn migration (Table 2). In spring, red deer located in areas with the largest spatial heterogeneity in snow cover duration had a higher probability to migrate (P = 0.82) compared to red deer located in areas with the smallest spatial heterogeneity in snow cover duration (P = 0.39; Table 2). In autumn, red deer located in areas with the lowest predictability in snow cover duration in their summer range had a lower probability to migrate (P = 0.16) than those located in areas with the highest predictability (P = 0.44; Table 2), although the effect was weak (small marginal R²).

**Table 2:** Candidate generalized linear mixed models (GLMM) to investigate the probability to migrate in spring or autumn in relation to the snow duration or the greenup/senescence dates at different spatio-temporal scales in five red deer populations.

Model	df	AICc	ΔAICc	wi	β	SE	Marginal R²/Conditional R²
***Spring (Winter to summer)***							
Snowmelt_large_	3	33.12	0.00	0.82	457.23	33.45	0.65/1.00
Snowmelt_predi_	3	36.83	3.71	0.13	64.71	23.39	—
Greenup_large_	3	39.66	6.54	0.03	222.21	55.04	—
Snowmelt_local_	3	41.24	8.12	0.01	2.27	0.58	—
Greenup_predi_	3	43.09	9.97	0.01	142.52	33.24	—
1	2	44.43	11.31	0.00	—	—	—
Greenup_local_	3	46.52	13.40	0.00	0.18	0.43	—
***Autumn (Summer to winter)***							
Snowfall_predi_	3	31.15	0.00	0.57	117.08	35.92	0.07/1.00
Senescence_local_	3	33.74	2.60	0.16	3.30	0.17	—
Senescence_predi_	3	34.08	2.93	0.13	236.04	0.00	—
Senescence_large_	3	34.41	3.26	0.11	372.38	47.78	—
Snowfall_local_	3	37.69	6.55	0.02	2.28	0.44	—
1	2	38.51	7.36	0.01	—	—	—
Snowfall_large_	3	41.67	10.52	0.00	78.27	24.30	—

Marginal and conditional R² are provided for models with ΔAICc < 2 to indicate model fit quality.

df = degrees of freedom; AICc = Akaike Information Criterion for small sample size; ΔAICc = Difference in AICc; wi = model weight; β = parameter estimate; SE = standard error of the parameter estimate.

**Figure 2:**
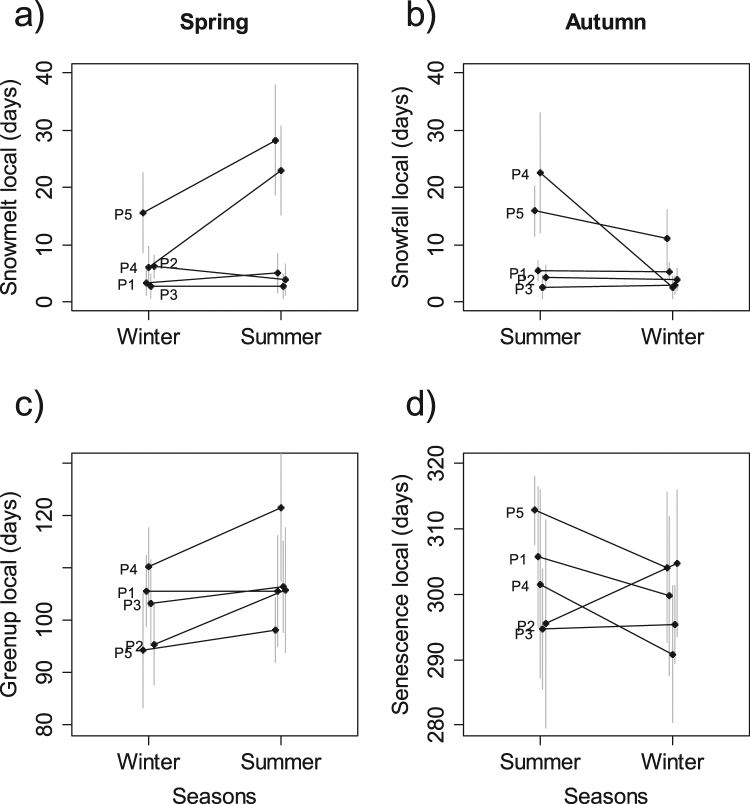
Mean (black dots) and standard deviation (gray bars) of local environmental conditions in the winter and summer ranges of the five red deer populations (P1 to P5). Snow cover duration after the 15^th^ January (**a**) and before the 15^th^ January (**b**). Greenup (**c**) and senescence (**d**) dates of vegetation based on NDVI. The first column represents the movements between winter and summer ranges (spring) and the second column represents the movements between summer and winter ranges (autumn). Black points: mean; grey segments: standard deviation.

We found highly significant differences in local-scale snow-cover duration across the five populations of red deer and between seasons. The snow cover duration was lower in the winter range than in the summer range for populations P4 and P5 which had the larger spatial heterogeneity in snow cover duration (Fig. 2; snowmelt_local_: season × population: df = 4, F = 20.72, P < 0.001; snowfall_local_: season × population: df = 4, F = 32.38, P < 0.001). However, in terms of greenup dates, there was no significant two-way interaction between season and population (df = 4, F = 1.49, P = 0.21), but the plant phenology was different between seasons (df = 1, F = 9.33, P = 0.003; Fig. 2) and among populations (df = 4, F = 12.53, P < 0.001; Fig. 2) meaning that greenup dates were constantly earlier in winter ranges compared to summer ranges in all populations, with different average greenup dates among populations. We found significant two-way interaction between season and population for the senescence dates (df = 4, F = 3.86, P = 0.007), with later senescence dates in summer ranges for three populations (P5, P1 and P4), and found the opposite for P2 and no significant differences between seasons for P3 (Fig. 2).

Individuals from the two populations with the highest proportion of migrants (P5 and P4) thus had local conditions with shorter snow-cover duration in winter than in summer ranges, whereas snow cover conditions did not vary much in other populations (P1, P2 and P3) between seasonal ranges (Fig. 2a and b). The seasonal difference in the plant phenology was smaller and not consistent with the proportion of migrants (Fig. 2). Indeed, the two populations having the highest greenup and senescence amplitude were P2 and P4, presenting contrasting situations in terms of proportion of migrants, from 0 to almost 60% of migrants, respectively.

*H2: Environmental factors affecting distance moved*

### Migrant individuals

**Figure 3:**
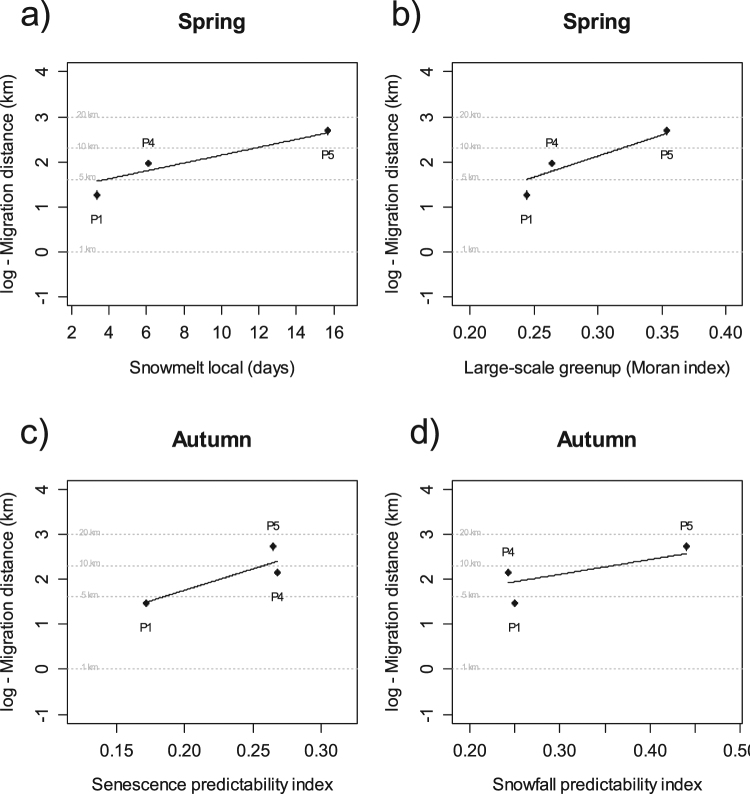
Mean and standard error (dots and bars) of the log-transformed migration distance in three populations of red deer in relation to (**a**) snowmelt estimated at the local scale and (**b**) greenup dates estimated at large scale for spring migration and in relation to (**c**) predictability of senescence dates and predictability of snowfall dates for the autumn migration (see text for details on explanatory variables). Lines represent prediction from linear mixed models for spring (a and b) and autumn (c and d).

In spring, the migration distance was strongly influenced by both local-scale snow cover duration and large-scale heterogeneity of greenup (snowmelt_local_ and greenup_large_, respectively; Table 3). These two variables were highly correlated (see Supplementary Table S3). The longer the duration of snow cover at local-scale and the higher the gradient of greenup dates at large-scale, the higher the distance moved by individuals from winter to summer ranges (Fig. 3). When local snow cover duration increased by 11.5 days (maximum amplitude; range = 4.5–16.0 days) in the last half of winter or when spatial heterogeneity of greenup dates increased by 0.11 (maximum amplitude; range = 0.24–0.35), migration distance increased by 11.8 km in average.

**Table 3:** Candidate linear mixed models (LME) to investigate the log-transformed migration distances in relation to the snow duration or the greenup/senescence dates at different spatio-temporal scales in the three red deer populations with migrant individuals.

Model	df	AICc	ΔAICc	wi	β	SE	Marginal R²/Conditional R²
**Spring (Winter to summer)**							
Snowmelt_local_	4	6.01	0.00	0.59	0.09	0.02	0.65/0.99
Greenup_large_	4	6.92	0.91	0.37	9.34	2.34	0.62/0.99
Greenup_local_	4	13.98	7.97	0.01	−0.04	0.02	—
1	3	14.36	8.35	0.01	—	—	—
Greenup_predi_	4	14.59	8.57	0.01	2.93	1.53	—
Snowmelt_large_	4	14.68	8.67	0.01	8.70	4.64	—
Snowmelt_predi_	4	17.11	11.09	0.00	1.33	1.37	—
**Autumn (Summer to winter)**							
Senescence_predi_	4	5.71	0.00	0.44	9.44	3.06	0.45/0.99
Snowfall_predi_	4	7.10	1.39	0.22	3.28	1.22	0.44/0.99
Senescence_large_	4	8.04	2.33ss	0.14	6.27	2.58	—
1	3	8.66	2.95	0.10	—	—	—
Senescence_local_	4	10.04	4.33	0.05	0.05	0.03	—
Snowfall_local_	4	11.14	5.43	0.03	0.03	0.02	—
Snowfall_large_	4	12.71	7.00	0.01	1.35	1.81	—

Marginal and conditional R² are provided for models with ΔAICc < 2 to indicate model fit quality.

df = degrees of freedom; AICc = Akaike Information Criterion for small sample size; ΔAICc = Difference in AICc; wi = model weight; β = parameter estimate; SE = standard error of the parameter estimate.

In autumn, the models with predictability in plant senescence and snow cover duration (senescence_predi_ and snowfall_predi_, respectively) at large scale received support to explain migration distance between summer and winter ranges (Table 3). The more predictable the senescence date and snow cover duration were, the longer the migration distance (Fig. 3). When senescence predictability increased by 0.10 (maximum amplitude; range = 0.17–0.27) the migration distance increased by 2.6 km. Similarly, when predictability of snow cover duration increased by 0.20 (maximum amplitude; range = 0.24–0.44) the migration distance increased by 5.7 km in average. Note that these results only rely on migrant individuals from 3 out of 5 populations, thereby reducing the statistical power of this analysis.

### Resident individuals

**Figure 4:**
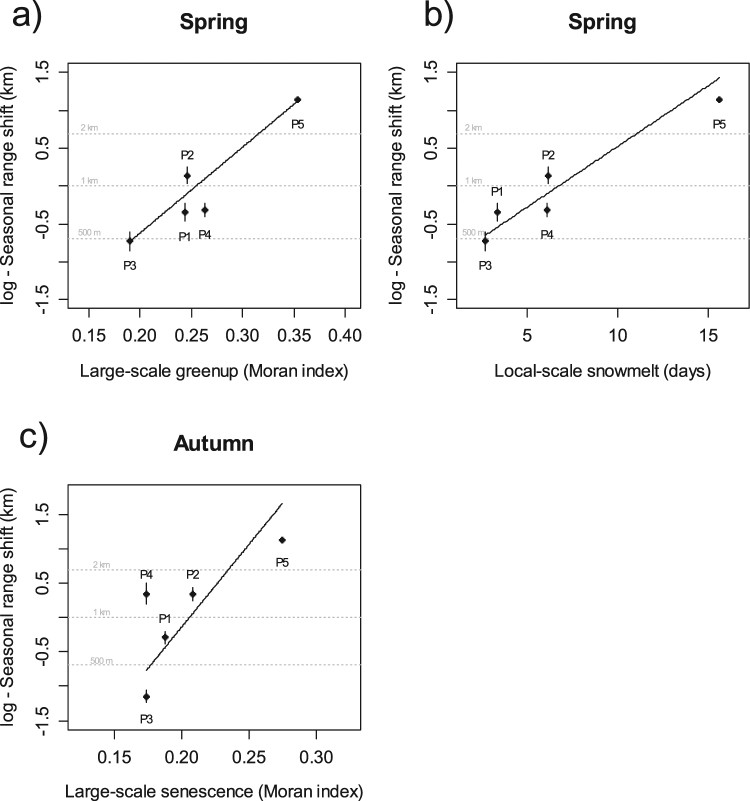
Mean and standard error (dots and bars) of the seasonal range shift in five populations of red deer. Winter to summer range shift in relation to (**a**) heterogeneity of greenup dates estimated at large scale and (**b**) snowmelt estimated at the local scale. Summer to winter range shift in relation to (**c**) heterogeneity of senescence dates estimated at large scale (see text for details on explanatory variables). Lines represent prediction from linear mixed models for spring (a and b) and autumn migration (c).

In resident individuals, seasonal range shifts between winter and summer ranges was explained almost equally by one large-scale variable and one local-scale variable (Table 4). First, an increase of 0.16 (maximum amplitude; range = 0.19–0.35) of spatial heterogeneity in greenup dates at large scale (greenup_large_) induced an increase of 2.64 km in seasonal range shifts from winter to summer ranges (Fig. 4a). Second, an increase of 13 days (maximum amplitude; range = 2.7–15.6 days) in local snow cover duration in winter (snowmelt_local_) induced also an increase of 2.64 km in seasonal range shifts from winter to summer ranges (Fig. 4b).

**Table 4:** Candidate linear mixed models (LME) to investigate the seasonal range shift in relation to the snow duration or the greenup/senescence dates at different spatio-temporal scales in five red deer populations.

Model	df	AICc	ΔAICc	wi	β	SE	Marginal R²/Conditional R²
***Spring (Winter to summer)***							
Snowmelt_local_	4	104.12	0.00	0.61	0.16	0.04	0.36/0.69
Greenup_large_	4	105.31	1.19	0.34	11.34	2.74	0.33/0.65
Greenup_local_	4	110.48	6.36	0.03	−0.09	0.03	—
Greenup_predi_	4	110.96	6.48	0.02	6.80	2.15	—
Snowmelt_large_	4	114.73	10.61	0.00	1.93	0.83	—
1	3	117.33	13.21	0.00	—	—	—
Snowmelt_predi_	4	118.13	14.02	0.00	1.55	1.18	—
***Autumn (Summer to winter)***							
Senescence_large_	4	163.28	0.00	0.98	24.05	4.57	0.35/0.44
Snowfall_local_	4	173.14	9.87	0.01	0.08	0.02	—
Senescence_local_	4	174.11	10.83	0.00	0.09	0.3	—
Snowfall _predi_	4	175.18	11.90	0.00	6.35	2.07	—
Senescence_predi_	4	177.61	14.33	0.00	8.00	3.10	—
Snowfall_large_	4	178.03	14.75	0.00	4.33	1.74	—
1	3	181.69	18.41	0.00	—	—	—

Marginal and conditional R² are provided for models with ΔAICc < 2 to indicate model fit quality.

df = degrees of freedom; AICc = Akaike Information Criterion for small sample size; ΔAICc = Difference in AICc; wi = model weight; β = parameter estimate; SE = standard error of the parameter estimate.

In autumn, seasonal range shifts between summer and winter ranges in resident red deer were strongly influenced by only one environmental characteristic, the large-scale gradient of senescence dates (senescence_large_; Table 4). An increase of 0.10 (maximum amplitude; range = 0.17–0.27) of spatial heterogeneity in senescence dates at large scale induced an increase of 2.80 km in seasonal range shifts (Fig. 4c).

The complementary analyses based on the percentage of seasonal range overlap, provided similar results and conclusions (Table S3; Fig. S2) as well as the analyses using different radii for the Moran index (Tables S5–S10). Overall, they showed that large scale variables influenced the seasonal movements even more as the Moran index was calculated over larger spatial scales.

## Discussion

Investigating populations heterogeneity in facultative migration patterns is an important step to understand processes and evolution of migration behaviors^9^. Our study included five West European populations of red deer. The contrast in seasonal migration among populations described in this study was based on a weak sample size and should be interpreted cautiously. However, with this in mind, we found important differences in average migration patterns among these populations. Although the maximum geographic distance between these populations did not exceed 500 km, the environmental conditions were very contrasted, from mountainous areas with varying snow cover to flat and more homogeneous environments. The probability for a red deer to migrate was strongly influenced by large-scale environmental conditions, as expected with H1. Regarding the seasonal movements characteristics, we found evidence that distances moved by both migrants and residents were related to large-scale environmental conditions as well, supporting H2. In this study, we showed that similar proximal causes influenced these seasonal movements, reinforcing the idea of a continuum from residency to migration in response to seasonal environmental changes.

The main finding of this study is that at the population scale, large-scale environmental conditions influence seasonal movements (both long- and short range movements) more than local-scale environmental conditions. Our results are consistent with the resource heterogeneity hypothesis predicting that environments with high spatial scaling (large-scale heterogeneity) favor migration^26^. In such environments, migration should result in effective habitat changes. In spring, animals may ride or jump the green wave to increase access to differential forage quality, following the time-lag in growing vegetation^18,21,30,31^. In autumn, animals can access areas with remnant vegetation where snow fall later in the season. Large-scale heterogeneity of resources not only affects the probability to migrate but also seasonal movements characteristics (migration distances in migrant individuals and seasonal range shifts in resident individuals). As expected, an increase in large-scale spatial heterogeneity of resource productivity induced longer migration distances in red deer to exploit the geographic gradients, at least during the spring migration. These results are not an artifact that could be due to varying seasonal range size as they were comparable among populations and the ratios between distances moved during migration (or seasonal range shifts) and seasonal range sizes also increased with large-scale heterogeneity. We also showed that large-scale heterogeneity of resource productivity increased seasonal range shifts in residents. The complementary analyses on seasonal range overlap led to the same conclusions, i.e. that large-scale heterogeneity of resource productivity reduced seasonal range overlap. At local-scale, only snow cover duration in spring influenced migration distances and seasonal range shifts, probably as it was highly correlated with greenup characteristics at large scale (the longer the snow cover duration, the larger the magnitude of the greenup date gradient at large scale).

Contrary to what could be expected from literature^23^, the proportions of migrants per population did not directly follow the latitude range of the populations, as the northernmost populations (P1 and P2) had only 25% and 0% of migrating red deer, respectively. This difference may be explained by different altitudinal gradients. Population P2, although at higher elevation than P1, had lower elevation amplitude (120 m *versus* 200 m for P2 and P1, respectively). This reinforces the idea that large-scale environmental conditions are more influential than local-scale environmental conditions on migratory tactics. The population P5 which exhibits the highest amplitudes in environmental conditions (with an elevation amplitude of 1000 m) had the highest proportion of migrants, the longest distances moved by migrants and the largest seasonal range shifts in residents. P4, the second population with the highest proportion of migrants also had the same elevation range, but was located at a lower overall elevation (max 1400 m *versus* 1800 m for P4 and P5, respectively).

The probability to migrate was more influenced by large-scale heterogeneity in snow cover duration, whatever the season (supporting H1b for autumn but not H1a for spring), than by plant phenology, although the results should be interpreted cautiously in autumn as the marginal R² remained weak (0.07; Table 2). In autumn, snow cover duration or snow depth have been shown to influence migratory movements in several large herbivore species such as moose in Scandinavia^23^, or roe deer in Norway^29^. In presence of extreme and predictable winter conditions, animals generally move from high elevation range with higher snow depth and/or snow cover duration to low elevation range with lower snow depth and duration when the winter is coming^32^. As such, access to forage resources is facilitated and vegetation remains accessible for a longer period of time.

Partially supporting H2, both large-scale and local-scale resource heterogeneity influenced the distances moved by migrants and residents in spring. Indeed, the large-scale heterogeneity in plant phenology was as important as local snow cover duration in explaining distances moved, both for migrants and residents (partially supporting H2a and H2b). Due to the key-role of food resources for ungulates during this season and as these two measures were highly correlated, these results were mainly supporting an influence of resource productivity on the distances moved by migrants and residents in spring. In autumn however, only large-scale resource predictability influenced the distances moved by all individuals, both large-scale heterogeneity in senescence dates and snow cover duration for migrants and only large-scale heterogeneity in senescence dates for residents (partially supporting H2c and not supporting H2d). Populations of mountainous environments (P4 and P5) displayed the longest migration movements to access areas with earlier emerging vegetation providing highly digestible forage^18,33^ in spring or late vegetation senescence in autumn. Among the other populations, those with a larger gradient of greenup dates in spring or senescence dates in autumn had longer migration distances than those with lower spatial scaling for these variables. However, in spring, high snow cover duration within initial ranges also stimulated both residents and migrants to move further from their initial range compared to individuals with low snow cover duration in our study areas.

In residents, the seasonal range shifts did not directly follow elevation range, except that the population with the largest seasonal range shifts is the Alps (P5) population, one of the populations with the highest elevation amplitude. On the contrary, the Jura population (P4), the second population with large elevation amplitude, displayed low seasonal range shifts. For this population, in spring, although the spatial greenup gradient is relatively high, the greenup predictability is low and both variables equally influenced seasonal range shifts. This suggests that when the environment is predictable, resident individuals shift their seasonal range to access higher quality vegetation. However, when the phenology is not predictable, it may be less advantageous for individuals to shift their range in areas with unknown resource quality. In autumn, only the large-scale heterogeneity in vegetation senescence influenced the seasonal range shifts, suggesting that the vegetation depletion and deterioration led individuals to shift their utilization distribution (UD), even if the senescence dates are not predictable. This could represent a leave it response (from the “take it or leave it” emergency life history strategy^34^) to the direct perturbation posed by this depletion and deterioration of the current range.

Snow cover duration is thus the trigger to long distance movement (migratory movements), but then the distance moved for both migrants and residents depends mainly on plant phenology. Our results therefore confirm that both migration and variation in seasonal range shifts in resident individuals are partly driven by the same factors. To our knowledge, these environmental drivers have never been investigated within a common framework for migrants and residents. Our results thus extend the findings from Cagnacci *et al*.^9^ on partial migration, where the authors defined migratory and residency tactics as a behavioral continuum. We broadened the concept of continuum but on drivers that lead to long and short seasonal movements (seasonal migration and short seasonal range shifts). Cagnacci *et al*.^9^ described the continuum in migration behaviors but only considering individuals that initiated migration (i.e. non-overlapping seasonal range). In this study, we highlighted the common triggers of both movements’ tactics.

In a context of global warming, animals may respond differently in spring and autumn. An early spring may change the average greenup date, but not its spatial structure. This would therefore not change the spring migration patterns in space (e.g., similar distance or shifts) but only in time (e.g., date, duration) as animals should follow the same resource gradient, but earlier^35,36^. Spring migration is generally related to parturition dates to match the peaks of high quality food, and although closely related species like roe deer lack plasticity in birth timing to adapt to earlier spring^37^, red deer was shown to adapt its breeding phenology to environmental changes^38^. On the other hand, global warming may also reduce snow depth and snow cover duration, which may affect distances moved from a seasonal range to the next. In addition, less snow-fall within summer ranges may directly reduce autumn seasonal movements as large-scale environmental heterogeneity decreases. Together, our findings suggest that global warming, by homogenizing large-scale environmental conditions may thus decrease migratory tactics. Alternatively, these landscape-level changes may influence the demography of the populations, increasing survival and/or recruitment of resident tactics compared to migratory tactics because the benefits from migration would decrease^1^. Ultimately, this would therefore lead to more individuals staying within the summer range (i.e. at high elevation in areas with high elevation range), then increasing the prevalence of the residency tactic in the population, with varying seasonal range shifts to adjust for small seasonal changes and decrease the risk of over-exploitation of food resources.

While drivers of migration have been widely investigated, little is known about the influence of the spatio-temporal gradients of snow cover and plant phenology on seasonal movements. This transversal study provided a better understanding of the causes and patterns of seasonal movements within the behavioral continuum migrants – residents. This study is the first to report empirical evidence that large-scale environmental heterogeneity affects all individuals within this continuum in a large herbivore species. Further investigation should now focus on the relationship between seasonal response intensity (migration – residency continuum and modulation of the distance moved) and the scale at which resource heterogeneity occurs.

## Material and Methods

### Study areas

Field work was carried out in five red deer populations. Two from Belgium (Hertogenwald and Saint Hubert populations, P1 and P2, respectively), one from France (La Petite Pierre, P3), one from Switzerland (Alps, P5) and on each side of the France - Switzerland border (Jura, P4), ranging between latitude 46.4°N and 50.6°N (Fig. 1). Study areas covered a high elevation range (from 250 to 2000 m) and varying landscapes, from deciduous to coniferous forest mixed with pastures and alpine grasslands (Table 1). Roe deer and wild boar coexist with red deer in all the study areas but chamois is also present at high elevation in Jura and Alps populations. All red deer populations were hunted during autumn and the beginning of winter in order to limit their densities. Natural predators were absent from Hertogenwald, Saint Hubert and La Petite Pierre populations but the lynx (*Lynx lynx*) was present in Jura (P4) and lynx and gray wolf (*Canis lupus*) in Alps (P5) populations,.

### Red deer location data

Red deer females were caught using drive netting (P1, P2 and P3), trapped (P3 and P4) or darted (P5). They were equipped with GPS collars (VECTRONICS in populations P1, P2, P4 and P5, and LOTEK in P3 and P4) mostly between 2009 and 2013 (Table 1). The median individual monitoring duration was equal to 346 days (range: [88; 1084]). The sampling regime of GPS locations was homogenized among study areas at 1 location every 4 hours. We considered 3D locations with a DOP < 5 and 2D locations with a DOP < 10 to keep the most accurate locations^39^. When the sampling regime was more intensive than 1 location every 4 hours, we selected the location with the lowest DOP at +/−60 min from the retained schedule: 0, 4, 8, 12, 16 and 20 h. This work was conducted in accordance with relevant national and international guidelines, and conforms to all legal requirements. Red deer captures and all experimental procedures were duly approved by legislation from the Wallon government (http://environnement.wallonie.be/legis/dnf/chasse/chasse028.htm) for populations P1 and P2, by the Prefecture of Paris (Prefectural Decree No. 2009–014) in agreement with the French Environmental Code (Art.R421-15 to 421-31 and R422-92 to 422-94-1) for population P3, by the Prefecture of Jura (prefectural decree no. 2011-1151) for the population P4 and by the Forest and Wildlife Service (canton of Fribourg), Forest, Wildlife and Nature Service (canton of Vaud), Agriculture and Nature Agency (canton of Berne) » for population P5. For all the populations, the methods were carried out in accordance with the approved guidelines and regulations.

We retained individual monitoring that covered at least two seasons (“winter”: 1st December to 28th February; “summer”: 1^st^ June to 31^st^ August) with at least 60 locations per season. Note that we excluded locations during the migratory movements per se to focus on seasonal home-ranges after settlement to avoid overestimation of home-range size due to migration relocation and to provide comparable home-range sizes between migrants and residents. Because facultative migration is widespread in large ungulates^19^, i.e. for an individual, seasonal movements a given year do not necessarily imply seasonal movements the next year and because we were interested in both spring and autumn movements independently, we did not impose a return phase to classify individuals as migrants. Thus, an individual was classified as migrant or resident considering home range shifts between two consecutive seasons only. We obtained monitoring for 52 individuals corresponding to 64 individual-years (the sampling unit in following statistical analyses) for the spring migration and 58 individuals corresponding to 76 individual-years for the autumn migration.

### Seasonal movements

For each individual-year, we estimated their winter (from 1^st^ of December to 28^th^ of February) and summer (from 1^st^ of June to 31^st^ of August) UDs from their location data. We used the same seasonal periods for all individuals to standardize the seasons and to facilitate comparisons among individuals and populations. We used kernel UD with a fixed smoothing level (h = 140), which provided a reliable way to compare utilization distributions among red deer^38^. For two consecutive UD (i.e., winter to summer or summer to winter) we computed the Volume of Intersection index (VI index, the volume that two UDs share in common, from 0 to 1^40^). We used these seasonal range overlap values to define as ‘migrant’ individuals having strictly no overlapping seasonal home-ranges and ‘resident’ individuals otherwise. We proceeded with a visual inspection of the raw location data to ensure there were no mistakes in the classification using time series of NSD, i.e. the squared distance between the first location and all the subsequent locations of an individual trajectory^41^. We also checked that migrant individuals monitored for more than two seasons came back to their initial range after migration by estimating the range overlap (using VI) between seasonal range i and seasonal range i + 2. We considered that individuals came back to their initial range when the VI index was >0.

For all individuals, we measured the linear displacement between consecutive seasonal geometric centres (spring: from winter to summer, autumn: from summer to winter). For residents only, we also used the individual range overlap (VI index) value to estimate the amplitude of seasonal range overlap, as complementary analyses presented in Supplementary Material. Indeed, the seasonal range overlap using the VI index represents a reliable spatially explicit method based on probabilistic estimates to assess change in home-range utilization^40^. These complementary analyses would account for the shape of the seasonal home-ranges. Two narrow seasonal ranges may be close to each other without overlapping much, meaning that despite the close proximity of the seasonal ranges, the individual completely changed its UD. Using the VI as complementary analyses for residents is therefore informative on the spatial behavior at stake. In addition, we estimated the ratio between seasonal movement distances and a measure of seasonal ranges’ size to provide information on the relative use of space for routine movements within seasonal ranges and the distance moved during migration. The seasonal ranges’ size was estimated by summing the radii of the initial seasonal range and the arrival seasonal range, considering a spherical shape of the ranges. A ratio close to one would mean that the distance moved during migration is relatively low compared to seasonal ranges’ size. The higher the ratio, the higher the distance moved during migration relative to seasonal range size.

Although we wanted to study the seasonal movement behavior continuum, we analyzed migration and residency separately, indexed by migration distances and seasonal range shifts, respectively. Indeed, our aim was to highlight potential differences or similarities in environmental characteristics responsible for both long (migrants) and short (residents) movements.

### Environmental conditions

We used the NDVI (MODIS satellite, 250 m resolution at 16-day intervals, https://modis.gsfc.nasa.gov) as a measure of the plant productivity^42,43^. For each pixel, we corrected annual NDVI dataset with a Best Index Slope Extraction (BISE^44^) algorithm, fitted double sigmoid curves (modeling an increase from a winter baseline to a summer plateau, then a return to another winter baseline) and derived “*greenup*” and “*senescence*” dates from curve coefficients.

We used a multi-annual snow cover dataset (MODIS satellite, 500 m resolution at 1 day intervals, (https://modis.gsfc.nasa.gov) that measures the daily presence/absence of snow. We summarized this dataset by calculating the cumulated number of days with snow present on the ground, both for the first half (15^th^ of July to 15^th^ of January) and the second half (15^th^ of January to 15^th^ of July) of each “winter”, referred to as *snowfall* and *snowmelt* thereafter.

We finally assigned these variables to each migratory season (e.g. *greenup* & *snowmelt* for spring migration season; *senescence* & *snowfall* for autumn migration season). Although elevation has been shown to influence migration in ungulates^25^, we did not include elevation as a co-factor as it was highly correlated with snow cover duration (R > 97%). All the environmental variables are summarized in Supplementary Table S1.

### Seasonal range characteristics

For following analyses, we needed estimates of environmental conditions for each individual in their initial range (i.e. on the winter range for spring migration and on the summer range for autumn migration) at two spatial scales: at local-scale and large-scale.

#### Local-scale conditions

The local-scale environmental conditions corresponded to the averaged snow cover duration and greenup/senescence dates within individual home-ranges. To provide standardized and comparable values of local-scale environmental conditions between home-ranges of varying size, we used a standardized buffer size. We first delimited the maximum available space for a given initial season (summer or winter ranges) for each individual-year with 100% minimum convex polygons (100% MCP) and estimated a mean radius among all individuals from their sizes (assuming circular home-ranges). This mean seasonal home-range size was 9.36 ± 12.19 km², resulting in an estimated mean radius of 1.73 km. We then delimited circular buffers with this radius, centred over each seasonal location geometric centre, named thereafter “seasonal range buffers” (see Supplementary Fig. S3). Within each seasonal range buffer, we then estimated greenup/senescence values averaged over all pixels (noted greenup_local_ and senescence_local_) and snow cover duration (noted snowmelt_local_ and snowfall_local_ for spring and autumn migration, respectively) by averaging all pixels of the seasonal range buffers. As we investigate seasonal movements at the population scale, these landscape variables were then averaged over all individuals within each population to obtain estimates of local-scale conditions at the population level.

#### Large-scale conditions

The large-scale environmental conditions corresponded to large-scale (1) heterogeneity and (2) predictability of environmental conditions in each study area (for each population). However, the capture protocols among populations were independently designed for a previous study and were thus heterogeneous, leading to both extensive and intensive monitoring of red deer in space. For example, the seven individuals of P5 were deliberately captured in different valleys, leading to individual locations covering an area of 1840 km², whereas important capture efforts (46 individuals) were made in the P3, within a restricted area of 120 km². The comparison of environmental heterogeneity in such contrasted areas was thus not relevant, as increasing areas mechanistically increases spatial heterogeneity of the environment. To standardize the scale of environmental measurements among populations, we therefore enlarged the seasonal range buffers’ radius until the 95% quantile of observed migration distance (equal to 7.171 km) to obtain “expanded buffers” for each individual (see Supplementary Fig. S3). We then averaged individual values for plant phenology and snow cover duration estimated on the expanded buffers to obtain population-level estimates at large-scale.

To estimate *large-scale heterogeneity of environmental conditions*, we computed Moran indexes^45^ on *greenup & snowmelt* for spring migration season and *senescence & snowfall* for autumn migration season on “expanded buffers”. Moran indexes are used to provide estimates of the variable’s heterogeneity, for a given distance. Moran indexes close to 1 or −1 indicated that neighboring pixels (below the threshold distance) were very similar or dissimilar, respectively, regarding more distant pixels of the expanded range (above the threshold distance). A value of ‘1’ thus corresponded to positive spatial autocorrelation (e.g. large-scale gradient), a value of ‘−1’ to a negative spatial autocorrelation (e.g. fine grained heterogeneity of resources) and values close to ‘0’ indicated no spatial structures (see an example in Fig. 4). To compute the indexes, we used the mean home-range radius as neighborhood threshold (i.e. 1.73 km), meaning that all pixels within the seasonal home-ranges were considered as close neighbors and those outside the home-range as distant neighbors. These variables representing spatial heterogeneity at large-scale were noted greenup_large_/snowmelt_large_ and senescence_large_/snowfall_large_ for spring and autumn migration, respectively. To test our approach at different spatial scales, we also computed the Moran indexes using radius x 0.5 and radius x 2 (see Supplementary Material Tables S4–S9).

To estimate *large-scale spatial predictability of environmental conditions*, we calculated the inter-annual spatial predictability of environmental conditions (snowmelt/snowfall and greenup/senescence dates) over expanded buffers. From multiannual datasets extracted on expanded buffers (minimum 6 years and maximum 10 years), we estimated the total spatio-temporal variance of *greenup & snowmelt* for spring migration season and *senescence & snowfall* for autumn migration season (among pixels and among years within pixels). Then we computed the mean inter-annual environmental conditions values for each pixel, estimated the variance of the resulting multiannual averaged map, and computed the ratio of this “spatial only” variance on the “total” variance as a measure of the spatial predictability. Values close to 1 indicated a high spatial predictability (spatial structures of environmental conditions were identical among years) and values close to 0 indicated a low spatial predictability (spatial structures of environmental conditions were highly different among years). These variables representing spatio-temporal predictability at large scale were noted greenup_predi_/snowmelt_predi_ and senescence_predi_/snowfall_predi_ for spring and autumn migration, respectively.

### Statistical analyses

We tested the influence of population, season and migratory status (migrant vs resident) on (1) log-transformed seasonal range surface area (estimated using 95% kernel) using linear mixed effects models (LME) with individual identity as a random effect on the intercept to control for repeated observations per individual, in particular for individuals monitored more than one year. Similarly, we tested the influence of population and migratory status on the ratio between seasonal movement distance and seasonal range size.

H1: To test the influence of local- and large-scale heterogeneity of environmental conditions on the probability to migrate (noted ‘1’) or to be resident (noted ‘0’), we used Generalized Linear Mixed Models (GLMM) with a binomial family and a logit link function and with individual identity as a random effect on the intercept to control for repeated observations per individual, as we had several individual-years per populations. Independently for each season, we then tested the influence of each environmental variable and different spatial scaling: greenup_local_, greenup_large_, greenup_predi_, snowmelt_local_, snowmelt_large_, and snowmelt_predi_ for spring seasonal movements; senescence_local_, senescence_large_, senescence_predi_, snowfall_local_, snowfall_large_, and snowfall_predi_ for autumn seasonal movements. As environmental variables took only five different values (five populations), the risk of over-parameterization was high (supporting artificially the more complex models). The candidate models thus included one variable only, leading to six candidate models in addition with a null model.

H1: To test differences in local-scale environmental conditions (snow cover duration and plant phenology within seasonal home-range) between seasons and populations, we used LME with individual identity as a random effect on the intercept to control for repeated observations per individual. We included a two-way interaction term between season and population to account for a possible differential effect of season among populations.

H2: To test for potential common drivers on seasonal movements between migrants and residents, we then separated the data into two classes. We used LME to test the influence of local- and large-scale environmental conditions on log-distance of migration for migrant individuals or on log-distance of seasonal range shifts for resident individuals. Similarly as for H1, each analysis (log-distance of migration and log-distance of seasonal range shifts) was performed separately for spring and autumn seasonal movements with candidate models including only one variable.

For all the analyses, we used the Akaike Information Criterion adjusted for small sample size (AICc) as recommended by Burnham and Anderson^46^ to select the best model. We retained the model with the lowest AICc value reflecting the best trade-off between complexity and precision. When the difference in AICc between two models (ΔAICc) was less than 2, models were considered as equivalents. We provided marginal and conditional R² for models with ΔAICc < 2 to indicate model fit quality^47^. We reported the correlation coefficient between explanatory variables in Supplementary Material (Tables S3 and Table S11). Analyses were performed using the package “adehabitatHR”^48^, “sp”^49^ and “nlme”^50^ from R software^51^.

### Data availability

All environmental GIS layers and location data generated from this study will be available as raster grids and R objects on Dryad.

## Electronic supplementary material

Supplementary information
