# Supplementary material for: Common drivers of seasonal movements on the migration – residency behavior continuum in a large herbivore
Source: Sci Rep. 2018 May 16;8:7631. doi: 10.1038/s41598-018-25777-y (PMC5956000; doi:10.1038/s41598-018-25777-y)
Supplement: Supplementary file 1 — Supplementary information [file 41598_2018_25777_MOESM1_ESM.docx]

**Supplementary Information**

Martin J., Tolon V., Morellet N., Santin-Janin H., Licoppe A., Fischer C., Bombois J., Patthey P., Pesenti E., Chenesseau D. & Saïd S. Common drivers of seasonal movements on the migration – residency behavior continuum in a large herbivore.

**Figure S1**: Net displacement of individual red deer misclassified as residents due to their seasonal range overlap value > 0% explained by short excursions from one seasonal range to the next or previous seasonal range. Blue dots represent winter ranges; green dots represent summer ranges. Misclassifications (resident instead of migrant) are indicated by a red line and an overlap value on the panels.

**Figure S2**: Mean and standard error (dots and bars) of the seasonal range overlap (estimated using Volume of Intersection index – VI index) in five populations of red deer. Winter to summer range overlap in relation to (a) predictability of greenup dates estimated at large scale, (b) snowmelt estimated at the local scale and (c) heterogeneity of greenup dates estimated at large scale (see text for details on explanatory variables). Summer to winter overlap in relation to local-scale senescence dates (d) (see text for details). Lines represent prediction from linear mixed models for spring (a, b, c) and autumn migration (d).


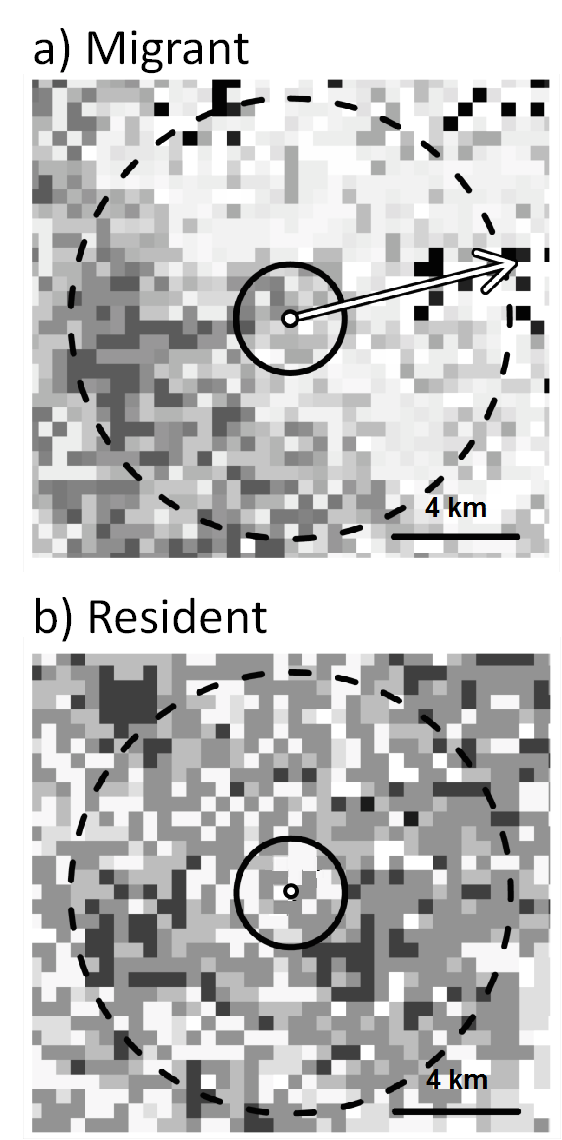


**Figure S3:** Conceptual example of two contrasted environmental conditions. Grids represent the gradient of environmental conditions (e.g. snow cover duration in number of day). a) Migrant individual in a population with large-scale heterogeneous conditions; b) resident individual in a population with low environmental heterogeneity. White points: seasonal range geometric centres. Black circles: seasonal range buffers (local-scale conditions); dashed circles: expanded buffers (large-scale conditions). The white arrow represents the potential migration distance and direction to the next seasonal range.

**Table S1**: Environmental conditions and spatial scales used to explain seasonal movements in red deer in spring (winter to summer ranges) and autumn (summer to winter ranges). Home-range buffer correspond to buffer with radius equal to the average radius of all individual-season home-ranges estimated using 100% minimum convex polygon; Expanded buffers correspond to enlarged home-range buffers up to 95% quantile of observed migration distance, to capture large-scale environmental conditions.

| **Name** | | **Variable** | **Scale** | **Measure** |
| --- | --- | --- | --- | --- |
| ***Spring*** | ***Autumn*** |  |  |  |
| Snowmelt _large_ | Snowfall _large_ | Snow cover duration | Expanded buffer | Spatial heterogeneity |
| Snowmelt _predi_ | Snowfall _predi_ | Snow cover duration | Expanded buffer | Spatial predictability |
| Snowmelt _local_ | Snowfall _local_ | Snow cover duration | Home-range buffer | Average values |
| Greenup _large_ | Senescence _large_ | Greenup/senescence dates of plants | Expanded buffer | Spatial heterogeneity |
| Greenup _predi_ | Senescence _predi_ | Greenup/senescence dates of plants | Expanded buffer | Spatial predictability |
| Greenup_local_ | Senescence _local_ | Greenup/senescence dates of plants | Home-range buffer | Average values |

**Table S2:** Ratio between the distances moved between consecutive seasonal ranges and the sum of the two respective radii. This ratio provides information on the distance moved in relation to the seasonal range size. The higher the ratio, the longer the distance moved compare to the seasonal range size.

|  | P1 | P2 | P3 | P4 | P5 |
| --- | --- | --- | --- | --- | --- |
| Migrants | 2.06 | - | - | 3.28 | 6.97 |
| Residents | 0.51 | 0.78 | 0.31 | 0.73 | 1.63 |

**Table S3**: Correlation between environmental variables in spring

|  | snowmelt_local_ | greenup_local_ | snowmelt_broad_ | greenup_broad_ | snowmelt_predi_ | greenup_predi_ |
| --- | --- | --- | --- | --- | --- | --- |
| snowmelt_local_ | 1 |  |  |  |  |  |
| greenup_local_ | -0.27 | 1 |  |  |  |  |
| snowmelt_broad_ | 0.69 | 0.49 | 1 |  |  |  |
| greenup_broad_ | 0.96 | -0.05 | 0.82 | 1 |  |  |
| snowmelt_predi_ | 0.55 | 0.57 | 0.89 | 0.63 | 1 |  |
| greenup_predi_ | 0.96 | -0.31 | 0.66 | 0.91 | 0.41 | 1 |

**Table S4**: Candidate linear mixed models (LME) to investigate the seasonal range overlaps (estimated using Volume of Intersection index – VI index) in relation to the snow duration or the greenup/senescence dates at different spatio-temporal scales in five red deer populations. We used this seasonal range overlap measure as complementary analyses. Indeed, distance between geometric centres for residents may be misleading as it does not consider the relative shape of the seasonal ranges. Two narrow seasonal ranges may be close to each other without overlapping much, meaning that despite the close proximity of the seasonal ranges, the individual completely changed its UD. However, our results revealed the same conclusions as for the distances between seasonal range geometric centres.

| **Model** | **df** | **AICc** | **ΔAICc** | **wi** | **β** | **SE** |
| --- | --- | --- | --- | --- | --- | --- |
| ***Spring (Winter to summer)*** |  |  |  |  |  |  |
| Greenup _predi_ | 4 | -23.33 | 0.00 | 0.41 | -1.25 | 0.43 |
| Snowmelt _local_ | 4 | -22.27 | 1.06 | 0.24 | -0.02 | 0.01 |
| Greenup _large_ | 4 | -22.09 | 1.24 | 0.22 | -1.57 | 0.58 |
| Greenup _local_ | 4 | -19.53 | 3.80 | 0.06 | 0.01 | 0.01 |
| Snowmelt _large_ | 4 | -18.13 | 5.20 | 0.03 | -0.30 | 0.17 |
| 1 | 3 | -17.31 | 5.48 | 0.03 | - | - |
| Snowmelt _predi_ | 4 | -15.86 | 7.48 | 0.01 | -0.19 | 0.25 |
| ***Autumn (Summer to winter)*** |  |  |  |  |  |  |
| Senescence _large_ | 4 | -41.82 | 0.00 | 0.85 | -4.10 | 0.86 |
| Snowfall _local_ | 4 | -36.58 | 5.24 | 0.06 | -0.01 | 0.00 |
| Snowfall _predi_ | 4 | -35.88 | 5.94 | 0.04 | -1.27 | 0.35 |
| Senescence _local_ | 4 | -35.02 | 6.81 | 0.03 | -0.02 | 0.01 |
| Senescence _predi_ | 4 | -33.50 | 8.33 | 0.01 | -1.70 | 0.54 |
| Snowfall _large_ | 4 | -31.91 | 9.92 | 0.01 | -0.88 | 0.31 |
| 1 | 3 | -26.54 | 15.28 | 0.00 | - | - |

df = degrees of freedom ; AICc = Akaike Information Criterion for small sample size; ΔAICc = Difference in AICc; wi = model weight; β = parameter estimate; SE = standard error of the parameter estimate.

**Variation of Moran Index estimation (Tables S5-S10)**

**Table S5:** Candidate generalized linear mixed models (GLMM) to investigate the probability to migrate in spring or autumn in relation to the snow duration or the greenup/senescence dates at different spatio-temporal scales in five red deer populations. Marginal and conditional R² are provided for models with ΔAICc < 2 to indicate model fit quality. Moran Index was calculated using radius / 2 (i.e. 36.5 km).

| **Model** | **df** | **AICc** | **ΔAICc** | **wi** | **β** | **SE** | **Marginal R²**  **/ Conditional R²** |
| --- | --- | --- | --- | --- | --- | --- | --- |
| ***Spring (Winter to summer)*** |  |  |  |  |  |  |  |
| Snowmelt _large_ | 3 | 31.17 | 0 | 0.93 | 362.2 | 107.2 | 0.52 / 1.00 |
| Snowmelt _predi_ | 3 | 36.83 | 5.66 | 0.05 | 64.71 | 23.39 |  |
| Greenup _large_ | 3 | 40.25 | 9.07 | 0.01 | 209.02 | 49.06 | - |
| Snowmelt _local_ | 3 | 41.24 | 10.07 | 0.01 | 2.27 | 0.58 | - |
| Greenup _predi_ | 2 | 43.09 | 11.92 | 0.00 | 142.52 | 33.24 | - |
| 1 | 3 | 44.43 | 13.25 | 0.00 | - | - | - |
| Greenup_local_ | 3 | 46.52 | 15.35 | 0.00 | 0.18 | 0.43 | - |
| ***Autumn (Summer to winter)*** | | | | |  |  |  |
| Snowfall _predi_ | 3 | 31.15 | 0.00 | 0.57 | 117.08 | 35.92 | 0.07 / 0.99 |
| Senescence _local_ | 3 | 33.74 | 2.59 | 0.16 | 3.30 | 0.17 | - |
| Senescence _large_ | 3 | 33.96 | 2.81 | 0.13 | 312.74 | 32.98 | - |
| Senescence _predi_ | 3 | 34.08 | 2.93 | 0.13 | 236.04 | 0.00 | - |
| Snowfall _local_ | 2 | 37.69 | 6.55 | 0.02 | 2.28 | 0.44 | - |
| 1 | 3 | 38.51 | 7.36 | 0.01 | - | - | - |
| Snowfall _large_ | 3 | 40.60 | 9.45 | 0.00 | 116.90 | 41.70 | - |

df = degrees of freedom ; AICc = Akaike Information Criterion for small sample size; ΔAICc = Difference in AICc; wi = model weight; β = parameter estimate; SE = standard error of the parameter estimate.

**Table S6:** Candidate generalized linear mixed models (GLMM) to investigate the probability to migrate in spring or autumn in relation to the snow duration or the greenup/senescence dates at different spatio-temporal scales in five red deer populations. Marginal and conditional R² are provided for models with ΔAICc < 2 to indicate model fit quality. Moran Index was calculated using radius x 2 (i.e. 146 km).

| **Model** | **df** | **AICc** | **ΔAICc** | **wi** | **β** | **SE** | **Marginal R²**  **/ Conditional R²** |
| --- | --- | --- | --- | --- | --- | --- | --- |
| ***Spring (Winter to summer)*** |  |  |  |  |  |  |  |
| Snowmelt _large_ | 3 | 35.07 | 0 | 0.62 | 87.08 | 27.00 | 0.65 / 0.99 |
| Snowmelt _predi_ | 3 | 36.82 | 1.75 | 0.26 | 64.71 | 23.39 | 0.14 / 1.00 |
| Greenup _large_ | 3 | 39.24 | 4.16 | 0.08 | 307.24 | 139.01 | - |
| Snowmelt _local_ | 3 | 40.95 | 5.88 | 0.03 | 2.27 | 0.58 | - |
| Greenup _predi_ | 3 | 43.08 | 8.01 | 0.01 | 142.52 | 33.24 | - |
| 1 | 2 | 44.44 | 9.37 | 0.01 | - | - | - |
| Greenup_local_ | 3 | 46.52 | 11.45 | 0.00 | 0.18 | 0.43 | - |
| ***Autumn (Summer to winter)*** | | | | |  |  |  |
| Snowfall _predi_ | 3 | 31.15 | 0.00 | 0.46 | 117.08 | 35.92 | 0.07 / 0.99 |
| Senescence _large_ | 3 | 32.16 | 1.02 | 0.28 | 513.86 | 58.70 | 0.04 / 1.00 |
| Senescence _local_ | 3 | 33.74 | 2.59 | 0.13 | 3.30 | 0.17 | - |
| Senescence _predi_ | 3 | 34.08 | 2.93 | 0.11 | 236.04 | 0.00 | - |
| Snowfall _local_ | 3 | 37.69 | 6.55 | 0.02 | 2.28 | 0.44 | - |
| 1 | 2 | 38.51 | 7.36 | 0.01 | - | - | - |
| Snowfall _large_ | 3 | 42.15 | 11.00 | 0.00 | 78.27 | 24.30 | - |

df = degrees of freedom ; AICc = Akaike Information Criterion for small sample size; ΔAICc = Difference in AICc; wi = model weight; β = parameter estimate; SE = standard error of the parameter estimate.

**Table S7:** Candidate linear mixed models (LME) to investigate the log-transformed migration distances in relation to the snow duration or the greenup/senescence dates at different spatio-temporal scales in the three red deer populations with migrant individuals. Marginal and conditional R² are provided for models with ΔAICc < 2 to indicate model fit quality. Moran Index was calculated using radius x 0.5 (i.e. 36.5 km).

| **Model** | **df** | **AICc** | **ΔAICc** | **wi** | **β** | **SE** | **Marginal R²**  **/ Conditional R²** |
| --- | --- | --- | --- | --- | --- | --- | --- |
| **Spring (Winter to summer)** |  |  |  |  |  |  |  |
| Snowmelt _local_ | 4 | 6.01 | 0.00 | 0.63 | 0.09 | 0.02 | 0.65 / 0.99 |
| Greenup _large_ | 4 | 8.10 | 2.09 | 0.22 | 8.26 | 2.24 | - |
| Snowmelt _large_ | 3 | 4.68 | 9.29 | 0.12 | 15.34 | 4.57 | - |
| Greenup _local_ | 4 | 13.98 | 7.97 | 0.02 | -0.04 | 0.02 | - |
| 1 | 4 | 14.36 | 8.35 | 0.01 | - | - | - |
| Greenup _predi_ | 4 | 14.59 | 8.57 | 0.01 | 2.93 | 1.53 | - |
| Snowmelt _predi_ | 4 | 17.11 | 11.09 | 0.00 | 1.33 | 1.37 | - |
| **Autumn (Summer to winter)** |  |  |  |  |  |  |  |
| Senescence _predi_ | 4 | 5.71 | 0.00 | 0.47 | 9.44 | 3.06 | 0.45 / 1.00 |
| Snowfall _predi_ | 4 | 7.10 | 1.39 | 0.24 | 3.28 | 1.22 | 0.44 / 1.00 |
| 1 | 3 | 8.66 | 2.95 | 0.11 | - | - | - |
| Senescence _large_ |  | 9.53 | 3.83 | 0.07 | 4.70 | 2.37 | - |
| Senescence _local_ | 4 | 10.04 | 4.33 | 0.05 | 0.05 | 0.03 | - |
| Snowfall _local_ | 4 | 11.14 | 5.43 | 0.03 | 0.03 | 0.02 | - |
| Snowfall _large_ | 4 | 11.67 | 5.97 | 0.02 | 3.35 | 2.69 | - |

df = degrees of freedom ; AICc = Akaike Information Criterion for small sample size; ΔAICc = Difference in AICc; wi = model weight; β = parameter estimate; SE = standard error of the parameter estimate.

**Table S8:** Candidate linear mixed models (LME) to investigate the log-transformed migration distances in relation to the snow duration or the greenup/senescence dates at different spatio-temporal scales in the three red deer populations with migrant individuals. Marginal and conditional R² are provided for models with ΔAICc < 2 to indicate model fit quality. Moran Index was calculated using radius x 2 (i.e. 146 km).

| **Model** | **df** | **AICc** | **ΔAICc** | **wi** | **β** | **SE** | **Marginal R²**  **/ Conditional R²** | |
| --- | --- | --- | --- | --- | --- | --- | --- | --- |
| **Spring (Winter to summer)** |  |  |  |  |  |  |  |  |
| Greenup _large_ | 4 | 3.76 | 0.00 | 0.75 | 21.51 | 4.43 | 0.70 / 0.99 |  |

| Snowmelt _local_ | 4 | 6.01 | 2.26 | 0.24 | 0.09 | 0.02 | - |
| --- | --- | --- | --- | --- | --- | --- | --- |

| Greenup _local_ | 4 | 13.98 | 10.23 | 0.00 | -0.04 | 0.02 | - |
| --- | --- | --- | --- | --- | --- | --- | --- |
| 1 | 3 | 14.36 | 10.60 | 0.00 | - | - | - |
| Greenup _predi_ | 4 | 14.59 | 10.83 | 0.00 | 2.93 | 1.53 | - |
| Snowmelt _predi_ | 4 | 17.11 | 13.35 | 0.00 | 1.33 | 1.37 | - |
| Snowmelt _large_ | 4 | 17.87 | 14.11 | 0.00 | -2.40 | 5.04 | - |
| **Autumn (Summer to winter)** |  |  |  |  |  |  |  |
| Senescence _predi_ | 4 | 5.71 | 0.00 | 0.44 | 9.44 | 3.06 | 0.45 / 0.99 |
| Snowfall _predi_ | 4 | 7.10 | 1.39 | 0.22 | 3.28 | 1.22 | 0.44 / 0.99 |
| Senescence _large_ | 4 | 8.44 | 2.73 | 0.12 | 11.36 | 4.92 | - |
| 1 | 3 | 8.66 | 2.95 | 0.10 | - | - | - |
| Senescence _local_ | 4 | 10.04 | 4.33 | 0.05 | 0.05 | 0.03 | - |
| Snowfall _local_ | 4 | 11.14 | 5.43 | 0.03 | 0.03 | 0.02 | - |
| Snowfall _large_ | 4 | 13.11 | 7.40 | 0.01 | 0.87 | 1.98 | - |

df = degrees of freedom ; AICc = Akaike Information Criterion for small sample size; ΔAICc = Difference in AICc; wi = model weight; β = parameter estimate; SE = standard error of the parameter estimate.

**Table S9:** Candidate linear mixed models (LME) to investigate the seasonal range shifts in relation to the snow duration or the greenup/senescence dates at different spatio-temporal scales in five red deer populations. Marginal and conditional R² are provided for models with ΔAICc < 2 to indicate model fit quality. Moran Index was calculated using radius x 0.5 (i.e. 36.5 km).

| **Model** | **df** | **AICc** | **ΔAICc** | **wi** | **β** | **SE** | **Marginal R²**  **/ Conditional R²** |
| --- | --- | --- | --- | --- | --- | --- | --- |
| ***Spring (Winter to summer)*** |  |  |  |  |  |  |  |
| Snowmelt _local_ | 4 | 104.12 | 0.00 | 0.56 | 0.16 | 0.04 | 0.36 / 0.69 |
| Greenup _large_ | 4 | 104.83 | 0.72 | 0.39 | 14.85 | 3.53 | 0.35 / 0.67 |
| Greenup _local_ | 4 | 110.48 | 6.36 | 0.02 | -0.09 | 0.03 | - |
| Greenup _predi_ | 4 | 110.96 | 6.84 | 0.02 | 6.80 | 2.15 | - |
| Snowmelt _large_ | 4 | 113.17 | 9.05 | 0.01 | 3.33 | 1.24 | - |
| 1 | 3 | 117.33 | 13.21 | 0.00 | - | - | - |
| Snowmelt _predi_ | 4 | 118.13 | 14.02 | 0.00 | 1.55 | 1.18 | - |
| ***Autumn (Summer to winter)*** |  |  |  |  |  |  |  |
| Snowfall _local_ | 4 | 173.14 | 0.00 | 0.32 | 0.08 | 0.02 | 0.18 / 0.53 |
| Senescence _large_ | 4 | 173.27 | 0.13 | 0.30 | 18.58 | 5.18 | 0.22 / 0.44 |
| Senescence _local_ | 4 | 174.11 | 0.97 | 0.20 | 0.09 | 0.3 | 0.20 / 0.59 |
| Snowfall _predi_ | 4 | 175.18 | 2.03 | 0.12 | 6.35 | 2.07 | - |
| Senescence _predi_ | 4 | 177.61 | 4.47 | 0.03 | 8.00 | 3.10 | - |
| Snowfall _large_ | 4 | 177.78 | 4.63 | 0.03 | 5.99 | 2.35 | - |
| 1 | 3 | 181.69 | 8.54 | 0.00 | - | - | - |

df = degrees of freedom ; AICc = Akaike Information Criterion for small sample size; ΔAICc = Difference in AICc; wi = model weight; β = parameter estimate; SE = standard error of the parameter estimate.

**Table S10:** Candidate linear mixed models (LME) to investigate the seasonal range shifts in relation to the snow duration or the greenup/senescence dates at different spatio-temporal scales in five red deer populations. Marginal and conditional R² are provided for models with ΔAICc < 2 to indicate model fit quality. Moran Index was calculated using radius x 2 (i.e. 146 km).

| **Model** | **df** | **AICc** | **ΔAICc** | **wi** | **β** | **SE** | **Marginal R²**  **/ Conditional R²** |
| --- | --- | --- | --- | --- | --- | --- | --- |
| ***Spring (Winter to summer)*** |  |  |  |  |  |  |  |
| Snowmelt _local_ | 4 | 104.12 | 0.00 | 0.85 | 0.16 | 0.04 | 0.36 / 0.69 |
| Greenup _large_ | 4 | 108.73 | 4.61 | 0.08 | 12.14 | 3.41 | - |
| Greenup _local_ | 4 | 110.48 | 6.36 | 0.03 | -0.09 | 0.03 | - |
| Greenup _predi_ | 4 | 110.96 | 6.48 | 0.02 | 6.80 | 2.15 | - |
| Snowmelt _large_ | 4 | 115.75 | 11.63 | 0.00 | 1.57 | 0.76 | - |
| 1 | 3 | 117.33 | 13.21 | 0.00 | - | - | - |
| Snowmelt _predi_ | 4 | 118.13 | 14.02 | 0.00 | 1.55 | 1.18 | - |
| ***Autumn (Summer to winter)*** |  |  |  |  |  |  |  |
| Snowfall _large_ | 4 | 169.78 | 0.00 | 0.44 | 6.72 | 1.67 | 0.28 / 0.54 |
| Senescence _large_ | 4 | 169.99 | 0.21 | 0.39 | 40.40 | 10.06 | - |
| Snowfall _local_ | 4 | 173.14 | 3.37 | 0.08 | 0.08 | 0.02 | - |
| Senescence _local_ | 4 | 174.11 | 4.33 | 0.05 | 0.09 | 0.3 | - |
| Snowfall _predi_ | 4 | 175.18 | 5.40 | 0.03 | 6.35 | 2.07 | - |
| Senescence _predi_ | 4 | 177.61 | 7.83 | 0.01 | 8.00 | 3.10 | - |
| 1 | 3 | 181.69 | 11.91 | 0.00 | - | - | - |

df = degrees of freedom ; AICc = Akaike Information Criterion for small sample size; ΔAICc = Difference in AICc; wi = model weight; β = parameter estimate; SE = standard error of the parameter estimate.

**Table S11**: Correlation between environmental variables in autumn

|  | snowfall_local_ | senescence_local_ | snowfall_broad_ | senescence_broad_ | snowfall_predi_ | senescence_predi_ |
| --- | --- | --- | --- | --- | --- | --- |
| snowfall_local_ | 1 |  |  |  |  |  |
| senescence_local_ | 0.88 | 1 |  |  |  |  |
| snowfall_broad_ | 0.85 | 0.66 | 1 |  |  |  |
| senescence_broad_ | 0.70 | 0.79 | 0.23 | 1 |  |  |
| snowfall_predi_ | 0.89 | 0.87 | 0.57 | 0.84 | 1 |  |
| senescence_predi_ | 0.97 | 0.80 | 0.93 | 0.52 | 0.84 | 1 |
